# A novel device for collecting and dispensing fingerstick blood for point of care testing

**DOI:** 10.1371/journal.pone.0183625

**Published:** 2017-08-24

**Authors:** Alexis F. Sauer-Budge, Samuel J. Brookfield, Ronald Janzen, Sarah McGray, Anna Boardman, Holger Wirz, Nira R. Pollock

**Affiliations:** 1 Center for Manufacturing Innovation, Fraunhofer USA, Brookline, Massachusetts, United States of America; 2 Department of Biomedical Engineering, Boston University, Boston, Massachusetts, United States of America; 3 PATH, Seattle, Washington, United States of America; 4 Boston Children's Hospital/Beth Israel Deaconess Medical Center, Boston, Massachusetts, United States of America; Holbæk Hospital, DENMARK

## Abstract

The increased world-wide availability of point-of-care (POC) tests utilizing fingerstick blood has led to testing scenarios in which multiple separate fingersticks are performed during a single patient encounter, generating cumulative discomfort and reducing testing efficiency. We have developed a device capable of a) collection of up to 100 μL of fingerstick blood from a single fingerstick by capillary action, and b) dispensing this blood in variable increments set by the user. We tested the prototype device both in a controlled laboratory setting and in a fingerstick study involving naive device users, and found it to have accuracy and precision similar to a conventional pipettor. The users also found the device to be easy to use, and recommended minor ergonomic improvements. Our device would allow performance of multiple POC tests from a single fingerstick blood sample, thus providing a novel functionality that may be of use in many testing settings worldwide.

## Introduction

Point-of-care (POC) diagnostics that utilize fingerstick (FS) blood are already in wide use in a range of clinical settings worldwide, and many more POC FS tests are currently in development. POC FS tests can lower the risk of loss-to-follow-up by enabling the clinician to make management decisions during the initial encounter with the patient, rather than requiring patients to return to the clinic for results. POC FS tests are also typically less expensive than tests done in central laboratories, providing advantages for testing in resource-limited settings. Importantly, it is frequently desirable to perform multiple FS tests for a given patient, such as those being evaluated for possible HIV treatment (e.g. HIV test, CD4 count, liver/kidney function, hemoglobin), for suspected malaria (e.g. malaria test, G6PD test, hemoglobin), or for routine antenatal care (e.g. HIV test(s), syphilis test, hemoglobin, blood typing). However, despite the explosion in production of new FS POC tests for these and other applications, little thought is being given to the inefficiency inherent in the performance of multiple separate FS tests on one individual during one clinical encounter, nor to the cumulative discomfort that those multiple FS inflict.

The practice of performing multiple POC tests for individual patients at a single visit is already widespread in some high volume primary care settings in resource-limited areas (e.g. [1–3]), and a recent study [3] clearly documented patient preference for FS (even that performed with blade-type lancets) over venipuncture. As the panel of available FS POC tests continues to expand (e.g. [4–8]), the anticipation is that the need to be able to do multiple FS tests in both asymptomatic (screening) and symptomatic (diagnosis) patients will grow substantially [9–11]. However, currently each POC FS test typically requires its own FS. In a study of implementation of FS-based POC testing for HIV anti-retroviral treatment initiation and monitoring in South Africa [1], the authors noted that in order to follow the South African HIV treatment guidelines, performance of recommended tests at POC would require each patient to have *up to 4 FS per visit*, in addition to the initial two FS needed for the HIV testing itself. Each of these POC diagnostics requires a specific volume (or volume range) of FS blood (typically ranging from 5 μL to 50 μL). Some products, having been validated with blood containing specific anti-coagulants (EDTA or heparin), have requirements for collection devices (e.g. capillary tubes) coated with these anti-coagulants; many of these platforms, however, allow the user to apply blood from uncoated capillary tubes as long as the blood is applied to the test device within a relatively short time frame (few minutes). Notably, while lancets that can elicit a large volume of blood (≥100 μL) from a single FS are commercially available and widely used ([1, 12–15]), it is not routinely possible to collect blood from that single FS with multiple collection devices in series; this creates infectious hazard (dripping blood), increases risk of blood coagulating during collection, and overall is physically challenging for the operator. In the study by Gous et al [1], up to 4 FS POC tests [CD4 count, creatinine (Cr), alanine aminotransferase (ALT), and hemoglobin (Hb)] were performed from one FS sample using multiple individual collection devices (capillary tubes and/or the test cartridge itself) in series; all POC tests were successfully completed from the single FS in 92% of patients (8% required a second FS). This study importantly confirmed that it was feasible to routinely collect ~100 μL blood from a single FS, that this collection method did not adversely impact the accuracy of the individual POC test results, and that the sequence of testing performed from that single FS did not impact results. However, the nurses required extensive training and experience in order to be able to execute the serial blood collection.

To address these issues, we have developed a device capable of collection and incremental dispensation of FS blood to enable the performance of multiple POC assays from one FS procedure. This device enables the user to collect up to 100 μL FS blood (from a single FS, performed with an appropriately-gauged lancet) using a single capillary tube, and to then serially dispense that blood in desired increments. Use of such a device could, in the future, offer the opportunity to reduce patient discomfort, reduce infectious hazard, and increase both diagnostic utility and acceptability of POC devices using FS blood. An additional long-term goal is for this device to be low-cost, given high potential for utility in resource-limited settings.

## Device design and methods

### Device description

**Fig 1:**
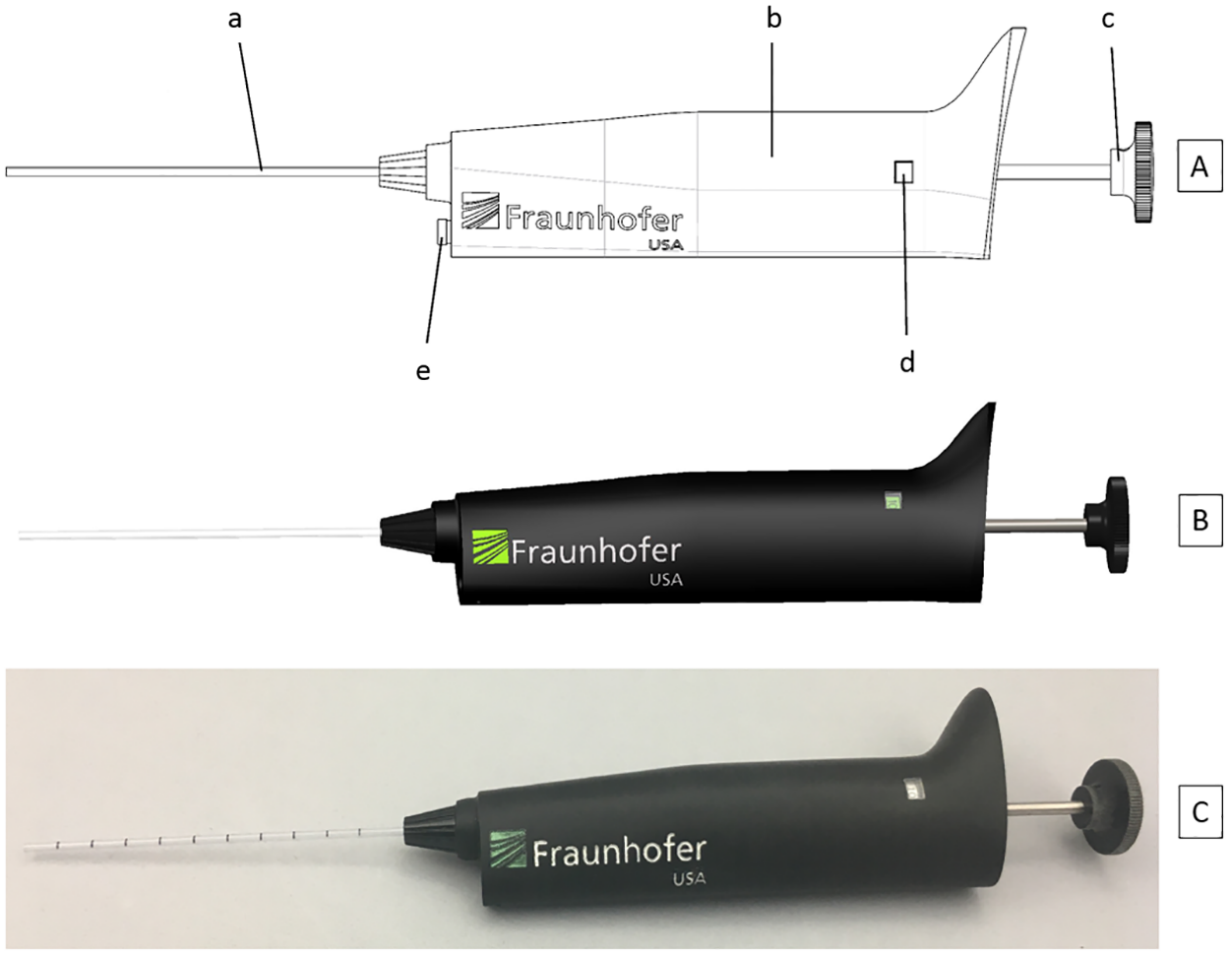
POC Blood Dispenser. Top (A): Sketch. [a] Capillary. [b] Housing. [c] Push button/volume adjustment knob. [d] Display window. [e] Open capillary indicator. Middle (B): 3D computer-aided design. Bottom (C): Prototype.

The POC Blood Dispenser accepts a capillary tube (Fig 1A, [a]) with a standard outer diameter of 1.9 mm (0.075”). It dispenses volumes between 5 μL and 50 μL in 5 μL increments. The device utilizes capillary action and air displacement to collect and dispense blood. Only the disposable capillary tube is in contact with blood; the dispenser does not contact the sample.

The handling and ergonomics (Fig 1) of the device are similar to a standard single-channel pipettor. The weight is 104.3 g and the length is 6.8” (without capillary tube). All operations are actuated by the push button/volume adjustment knob (Fig 1A, [c]). Turning the knob sets the desired dispense volume or switches to capillary filling mode. The setting is displayed in the window (Fig 1A, [d]). Pushing the button controls filling and dispensing blood from the capillary. The open capillary indicator (Fig 1A, [e]) appears at the bottom of the device when the capillary is unsealed and ready for filling.

The device’s custom parts are a combination of rapid prototyped parts (Stratasys Objet30 with VeroBlackPlus) and machined parts (stainless steel). It also uses commercially available hardware (screws, nuts, springs, o-rings, and dowel pins).

**Fig 2:**
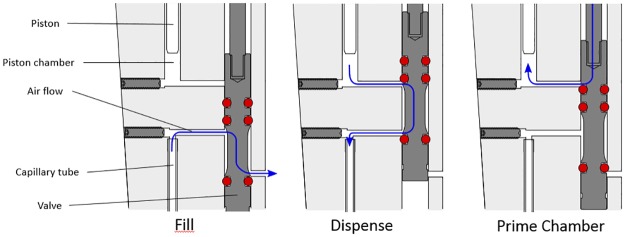
The shuttle valve in each of its three positions. Left: shuttle valve position one; airflow between capillary and atmosphere, allowing capillary to be filled. Middle: shuttle valve position two; airflow between piston chamber and capillary, allowing blood to be dispensed. Right: shuttle valve position three; airflow between atmosphere and piston chamber, allowing the chamber to be primed for another dispense.

The device has two key mechanisms that allow it to be both adjustable and repeating. One is the three-position shuttle valve (Fig 2), which opens and closes air passages as necessary to ensure proper device operation. The valve’s first position opens the capillary to the atmosphere, allowing blood to enter the tube via capillary action. In its second position, the valve opens the air passage between the capillary tube and the piston chamber, allowing blood to be dispensed from the capillary. As the piston (the position of which is controlled by the push button/volume adjustment knob) moves downward, it forces air through the open passage, in turn moving blood out of the capillary. The shuttle valve’s third position seals the capillary but opens the piston chamber to the atmosphere, so when the piston retracts after dispensing, air refills the chamber and the blood stays in place in the capillary. If there is blood in the capillary and the user has released the push button/volume adjustment knob, but is not ready to dispense (e.g. the capillary has just been filled with blood and the user has put down the Blood Dispenser to bandage the patient, or the user has dispensed one volume but wishes to pause before dispensing another), blood will not leak from the capillary because it is sealed from the top (just as a capillary will not leak if a finger is used to block one end).

**Fig 3:**
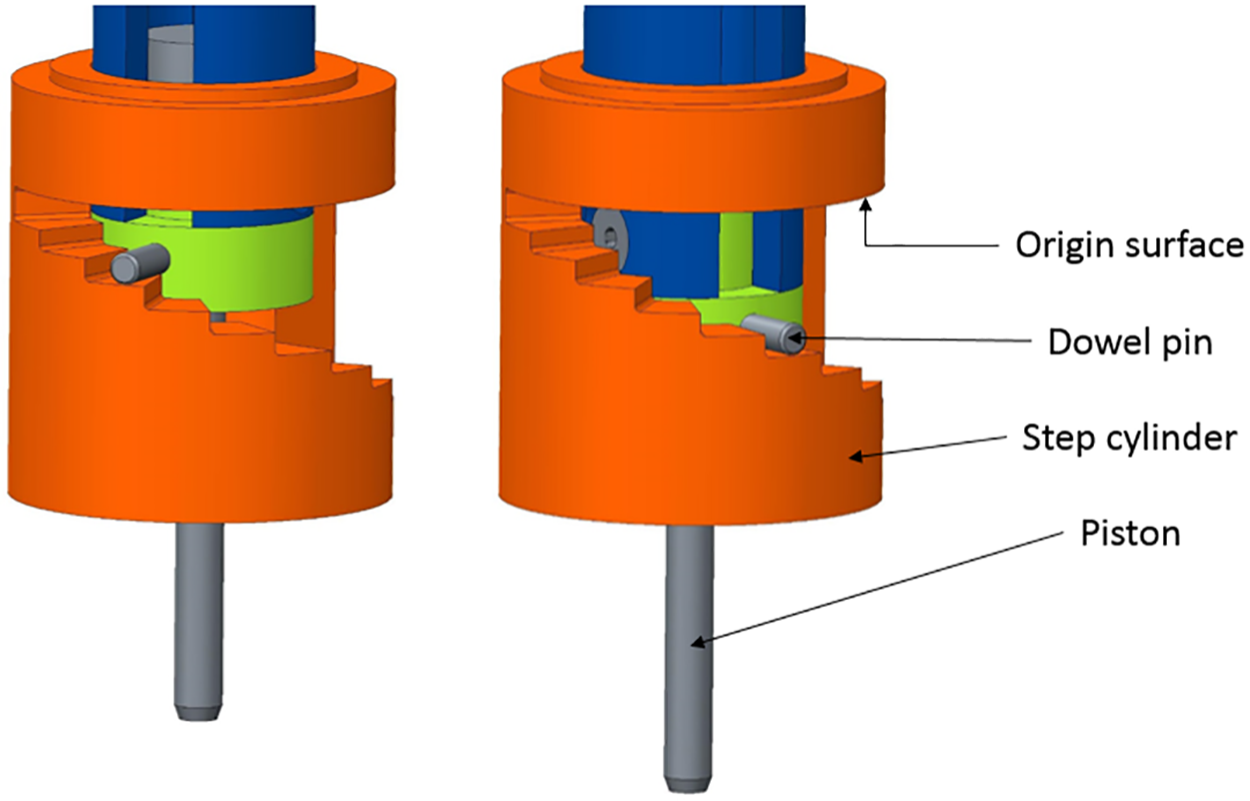
The volume adjustment mechanism. Left: dispensing 10 μL. Right: dispensing 25 μL.

The second key mechanism is the volume adjustment mechanism (Fig 3), which allows the dispense volume to be adjusted even while a sample is being held in the capillary tube by capillary action. The first component of this mechanism is the dowel pin, which moves with the push button/volume adjustment knob; when the user turns the knob, the dowel pin rotates to a different position, and when the user pushes the button, the dowel pin moves down. The second component is the step cylinder, which remains fixed despite movement of the other components. Each angular position of the dowel pin has a corresponding step that allows the dowel pin to move a different vertical distance from its origin surface. Each step corresponds to a different dispense volume. In total, there are 11 steps, with the shallowest allowing no dowel pin travel. The steps are machined to ±0.0005” tolerances from the origin surface to tightly control the travel of the dowel pin. These tight tolerances are crucial because the third component of the volume adjustment mechanism, the piston, which controls how much blood moves out of the capillary during each dispense event, travels the same distance as the dowel pin; thus, tightly controlling the dowel pin’s travel helps minimize potential systematic errors in dispensed blood volumes.

### Blood

For in-house testing at Fraunhofer CMI, K3 EDTA-treated whole blood purchased from Biological Specialty Corporation was used. For clinical testing at PATH headquarters, blood was collected from human donors using the POC Blood Dispenser (see “*FS Sample Performance Evaluation and Usability Testing*,” below).

### Filter paper

**Fig 4:**
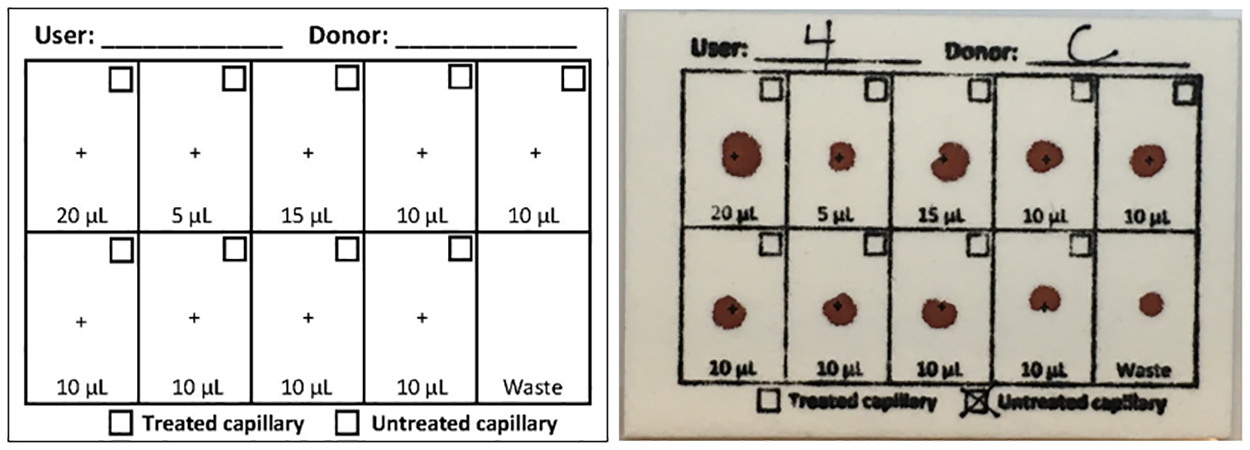
The Blood Collection Card (BCC), 7 x 10 cm. Left: BCC illustration. Right: completed BCC.

During both in-house and clinical testing, blood was dispensed onto thick, cotton chromatography paper (VWR International, Radnor, PA; P/N 28334–166), referred to here as the Blood Collection Card (BCC). In-house testing used the BCC with no modifications; for clinical testing and for development of an associated benchmark for volume measurement, the BCC (7 x 10 cm) was stamped with a custom design (Fig 4) indicating desired dispense volumes, target dispense areas (marked with “+”, with a box to mark if anything unusual occurred during the dispense event), and spaces to track device users (1–5), de-identified donors (A–D), and type of capillary used (treated or untreated, below).

### Capillary tubes

Much of the functional testing during the development phase was performed with glass capillaries (Kimble Chase, Vineland, NJ; P/N 71900–100). Plastic, 150 μL end-to-end capillaries, both untreated and sodium heparin-treated (Ram Scientific, Nashville, TN; P/Ns 060199 and 060007, respectively), were used for performance evaluation and usability testing and associated in-house volume benchmark development. For device and benchmark development, glass and plastic capillaries were marked at 100 μL, and for performance evaluation and usability testing, the plastic capillaries were marked in 10 μL graduations up to 100 μL.

### Device testing

#### In-house testing (Fraunhofer CMI)

The goals of in-house testing were to: 1) gain feedback on the device’s usability; 2) assess device accuracy both in the hands of an “experienced” user, who had been testing the device throughout the development process, and in the hands of two “new” users, who were given only minimal training and instruction prior to testing.

For in-house testing, the user placed a fresh BCC onto a scale and tared the scale. The user then put approximately 300 μL of blood into a disposable plastic tray to mimic blood pooling on a patient’s fingertip after a FS. With the POC Blood Dispenser, the user collected a blood sample (maximum 100 μL) from the tray, and proceeded to dispense 5, 10, 15, and 20 μL volumes 10 times each onto the BCC while it was on the scale, refilling the capillary as necessary. To collect volume accuracy data, the scale readout was recorded and tared between each dispense. To convert weight to volume, 1.060 mg/μL was used as the density of blood. Points of capillary refill were also recorded.

For comparison, we also weighed 10 blood samples each of 5, 10, 15, and 20 μL dispensed with a commercial P20 pipette (Eppendorf, Hamburg, Germany; P/N 4920000032).

#### FS sample performance evaluation and usability testing (PATH)

**Fig 5:**
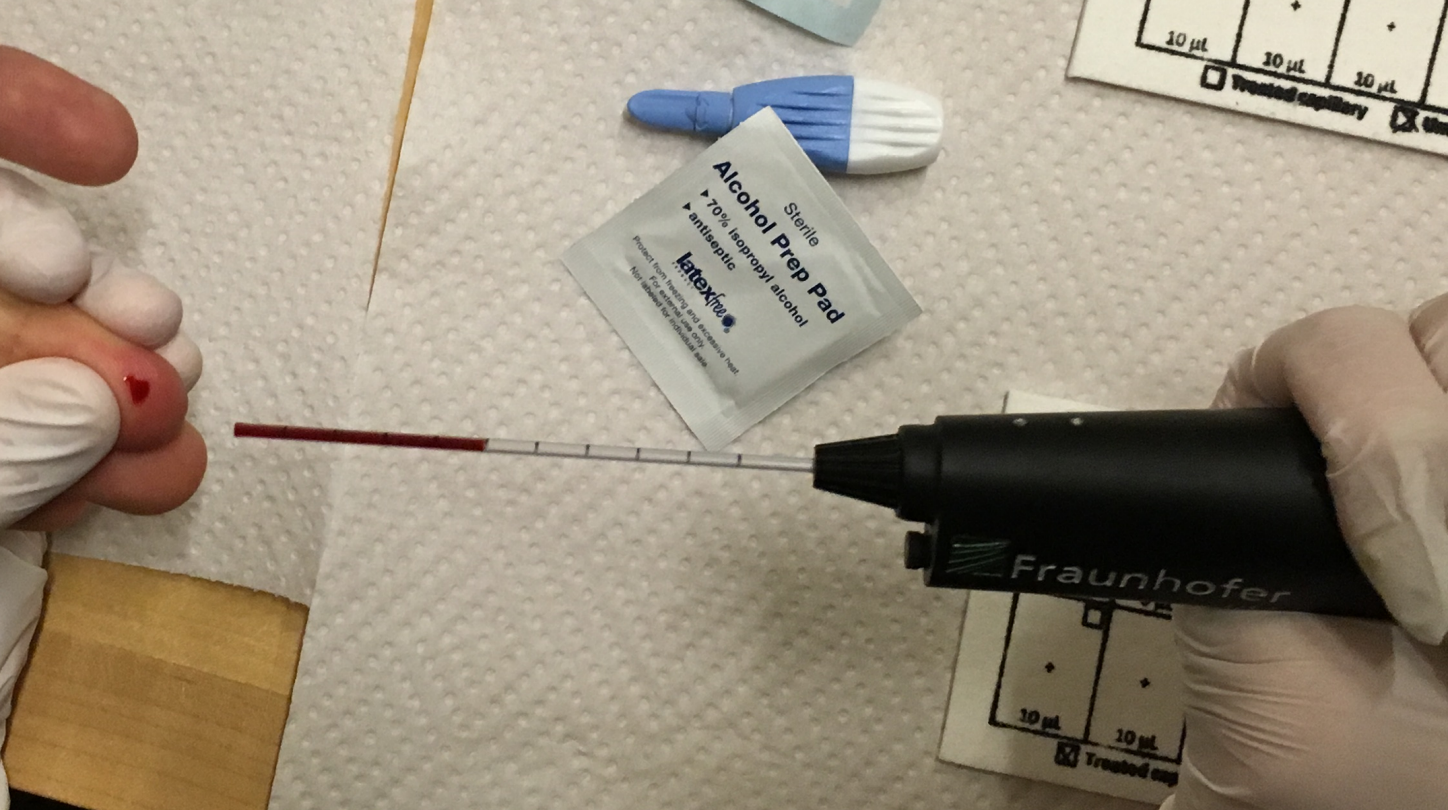
Blood being collected from donor with POC Blood Dispenser during clinical testing. May 26, 2016. PATH, Seattle, WA.

The device was evaluated at PATH, a global health nonprofit, to assess the functionality and usability of the POC Blood Dispenser in a simulated point-of-care setting, in terms of user requirements, ergonomics, and overall dispensing accuracy (Fig 5) using fresh FS whole blood specimens. Usability testing was conducted in a contrived environment at PATH as a first-pass verification of device usability, with the expectation of human factors validation in target use settings in later phases of product development. The testing was performed by a group of PATH staff volunteers with familiarity with the intended use environments (resource-constrained diagnostic settings), and was supervised by two Fraunhofer CMI staff members (S.J.B. and A.F.S.B.) and one PATH staff member (S.M.). The volunteer group consisted of five device users and 21 blood donors. The device users were variably familiar with FS procedures, but had never seen or used the POC Blood Dispenser prior to the testing day. The users received a brief training on the device before commencing with FS, blood collection, and blood dispensing. The evaluation was reviewed and approved by the PATH Research Ethics Committee. All participants provided verbal informed consent prior to participating in the evaluation.

The study was designed for each user to take two FS, performed with contact-activated lancets (BD, Franklin Lakes, NJ;P/N 366594), from each of four different blood donors. For each donor, blood from the first FS was collected with a sodium heparin-treated capillary loaded into the device, and blood from the second FS was collected with an untreated capillary loaded into the device. The user attempted to collect 100 μL of blood from each FS, and then to dispense as many increments of blood as possible onto the BCC, starting with 5 μL to “Waste”, then moving to the upper left hand corner and completing the top row first, followed by the remainder of the second row. As with any FS procedure, there is potential for blood drips to contaminate gloves, work surface, or the exterior of the device. Therefore, the device was disinfected with a bleach wipe between donors.

An observer recorded relevant times/intervals for each procedure: when blood began to be drawn into the capillary tube, the first aliquot onto the BCC, the completion of dispensing (empty capillary tube), and if/when blood coagulated in the capillary tube. The observer also recorded any notable events (e.g. air bubbles in the capillary tube, incomplete/wrong dispenses), and user feedback about that particular FS blood collection and dispensing event (S1 File). In addition, the event was captured on video, which was analyzed using MaxQDA qualitative analysis software. The videos were reviewed and coded for usability and use-error indicators, as well as related qualitative data presented during the exit interviews. Qualitative analysis was conducted by an expert in user experience research for resource constrained healthcare settings (S.M.). In addition, users were presented with pre/post questionnaires and interviewed at the conclusion of their participation (S1 File). The pre/post questionnaire presented three questions (two Likert-style scales and one A/B comparison) to quantitatively gauge users’ general perception of the utility and ease of use of the device and to compare perceptions prior to and after using the device. The exit interview captured qualitative feedback on the users’ experiences with the device to inform adaptations to the device design and instructions for use. At the conclusion of the study, each BCC was photographed in a StandScan lightbox (Standscan, Hong Kong) for later image analysis.

### Image analysis

ImageJ software (National Institutes of Health, Bethesda, MD) was used to conduct image analysis of the BCCs to estimate how accurately the device dispensed FS blood in the hands of new users during PATH testing. The goal of the image analysis was to correlate the number of pixels in a bloodspot to the volume of a bloodspot.

To establish this relationship, we created a benchmark using commercial P10 and P100 pipettes (Eppendorf, Hamburg, Germany; P/Ns 3120000020 and 3120000046, respectively) to dispense K3 EDTA-treated whole blood onto BCCs. Aliquots of 5, 10, 15, and 20 μL were each dispensed 10 times. We photographed the resulting bloodspots in the StandScan lightbox and analyzed the images using ImageJ. The red element from the RGB spectrum was isolated from the images, a process that simultaneously converted them to 8-bit grayscale images. Each image was then turned into a black and white image using the automatic threshold feature. Using the ROI and wand features, the pixel numbers for each blood spot were calculated in ImageJ. We plotted a best-fit curve through the average pixel numbers for each volume. We also noted the minimum, maximum, and standard deviation for each volume.

We performed the same image analysis on each of the BCCs from the FS performance evaluation to get the number of pixels for each bloodspot. A volume measurement for each blood spot was assigned using the benchmark best-fit curve.

## Results and discussion

### In-house testing

Table 1 shows the precision and accuracy of the POC Blood Dispenser in the hands of an experienced user compared to the commercial P20 pipettor, and the POC Blood Dispenser compares favorably: its systematic errors are smaller than those of the P20 for all volumes tested, and it has a smaller random error at 5 μL, with random errors as a percentage of the target volume ranging from 2.1–3.7% larger than the P20 for the other three volumes tested (“Comparison” rows). Note that the data for the POC blood dispenser do not count the first aliquot after the capillary had been filled. We discounted this initial aliquot (and similarly had users dispense the first 5 μL to waste during the performance evaluation) because preliminary testing revealed that the volume of the first aliquot dispensed after filling was consistently below average, and that the device essentially needed to be primed after filling to achieve best results (data not shown).

**Table 1:** In-house gravimetric testing results for the POC Blood Dispenser by an experienced user compared to in-house gravimetric testing results for a P20 pipettor.

Device/Standard	Measurement	Volume [μL]			
		5	10	15	20
POC Blood Dispenser	Mean	4.72	9.76	14.0	18.7
	Systematic Error	-0.28	-0.24	-1.02	-1.26
	Random Error	0.60	0.87	0.91	1.22
P20	Mean	4.56	8.96	13.8	18.3
	Systematic Error	-0.44	-1.04	-1.16	-1.68
	Random Error	0.94	0.58	0.59	0.48
Comparison	Systematic Error Difference	-0.16	-0.80	-0.14	-0.42
	Random Error Difference	-0.34	0.29	0.32	0.74

Measurements for both were taken on the same scale. P20 was calibrated one month prior to testing. Systematic error is defined as: $\bar{v}-v_{T}$, where $\bar{v}$ is the mean and v_T_ is the selected target volume. Random error is defined as: $\sqrt{\frac{1}{N-1}\sum_{i=1}^{N}{\left(v_{i}-\bar{v}\right)}^{2}}$, where v_i_ is each measurement, and N = 10 (10 data points at each target volume). Systematic Error Difference is the POC Blood Dispenser Systematic Error subtracted from the P20 Systematic Error. Random Error Difference is the P20 Random Error subtracted from the POC Blood Dispenser Random Error. Note that in the “Comparison” rows, a negative number indicates that the POC Blood Dispenser performed better than the P20, while a positive number means the opposite.

Results from the accuracy testing as performed by two new users are shown in Table 2, compared to the results from an experienced user. As with the experienced user data, the new user’s data do not include the first aliquot after the capillary had been filled. As expected, the errors are generally larger for new users than for an experienced user.

**Table 2:** In-house gravimetric testing results for the POC Blood Dispenser by two new users compared to the experienced user results from Table 1.

User Group	Measurement	Volume [μL]			
		5	10	15	20
Experienced User	Mean	4.72	9.76	14.0	18.7
	Systematic Error	-0.28	-0.24	-1.02	-1.26
	Random Error	0.60	0.87	0.91	1.22
New Users	Mean	4.37	8.13	12.8	19.8
	Systematic Error	-0.63	-1.88	-2.16	-0.21
	Random Error	0.55	1.37	1.24	0.84
Comparison	Systematic Error Difference	-0.35	-1.64	-1.14	1.05
	Random Error Difference	0.04	-0.50	-0.33	0.38

Systematic and random errors defined as in Table 1, except that here there are 20 total data points at each volume for the new users (10 data points for each of the two new users). Systematic Error Difference is the Experienced User Systematic Error subtracted from the New User Systematic Error. Random Error Difference is the New User Random Error subtracted from the Experienced User Random Error. Note that in the “Comparison” rows, a negative number indicates that Experienced Users performed better than New Users, while a positive number means the opposite.

### FS blood performance evaluation

On average, 89 μL of blood was collected from each FS. 90 μL or more of blood was collected in 33/42 draws (78.6%); less than 70 μL was collected in the 9 other cases. It took, on average, 2:24 (minutes:seconds) to complete the blood draw (i.e. from the initial puncture of the finger with the lancet to the first dispense of blood from the capillary tube). The time between the first dispense and the last dispense was, on average, 1:00, for a total average procedure time of 3:24.

Coagulation within the capillary tube occurred only twice during the FS performance evaluation, both times with an untreated capillary. The first dispenses in these two cases occurred 3:18 and 4:08 after the blood collection began, two of the four slowest times for the untreated capillaries (other two slowest times: 3:18 and 4:34).

Table 3 presents data for estimates of blood volumes dispensed during the FS performance evaluation. The systematic error as a percentage of the target volume generally decreased as volume increased: 17% error for 5 μL, 12% error for 10 μL, 0.2% error for 15 μL, and 5% for 20 μL. The random error as a percentage of the target volume is more stable: 22% for 5 μL, 13% for 10 μL, 19% for 15 μL, and 16% for 20 μL.

**Table 3:** Fingerstick blood testing results for the POC Blood Dispenser.

Measurement	Volume [μL]			
	5	10	15	20
Mean	5.84	11.2	15.0	19.0
Systematic Error	0.84	1.18	0.03	-0.95
Random Error	1.08	1.33	2.80	3.24

Given many limitations to the image analysis (e.g. blood spots differing in color due to differential duration of blood drying on BCCs prior to photography, changing ambient light conditions affecting image consistency, ink crosshairs on the BCCs impeding blood flow in a non-uniform manner, and crosshairs being only partially enveloped by dispensed blood), each of which in turn affected our estimates of dispensed volumes, the accuracy and precision data were not analyzed in further detail.

### Usability testing

Overall, the usability of the POC Blood Dispenser was good; users were able to properly operate the device with minimal training. The primary points of friction during device use were related to challenges drawing up sufficient quantities of FS blood into the capillary tube, which was highly dependent on the donor and on the technique used in conducting the FS. In exit interviews, users consistently noted that the device was “pretty easy” or “relatively easy” to use, although a learning period was required to become comfortable with it. This generally positive feedback is also reflected in the quantitative results of the Likert-style questions of the pre/post questionnaire, where users’ quantitative measures of perceived usability did not differ from before and after (4 out of 5 points in both cases). In addition, all users perceived the device as having greater utility over the status quo in pre- and post-questionnaires.

In-house testing and the FS performance evaluation revealed six opportunities to improve upon device usability. First, some users struggled with pressing the push button/volume adjustment knob through its full stroke when dispensing blood. During the FS performance testing, this error occurred during four attempted dispenses. When this occurred, the selected volume of blood was dispensed properly, but then an equivalent volume of air was sucked back into the capillary (this does not cause contamination). This is remedied by keeping the volume setting the same and pressing the button through its full stroke, bringing the fluid to the capillary tip again. The issue arises because there are small changes in resistance through the full stroke length due to the device’s mechanics, and users occasionally perceive one of these changes in resistance to be the end of the stroke length. This issue may be addressed in the next generation of design, either with mechanical adaptations or the addition of visual cues on the device.

Second, the plastic capillaries used in the performance evaluation were found to introduce a specific use-error that had not been seen with glass capillaries. For most of the testing during the device development phase, glass capillaries were used, but for the performance evaluation, we used plastic tubes, under the assumption that they would be preferable to avoid the risk of breaking a glass capillary tube during the filling and dispensing processes. Unexpectedly, we noted that in order to induce capillary action during the blood collection step, many users bent the flexible plastic capillaries to create the proper angle between the donor’s finger and the capillary, and on a few occasions, the bent tube “snapped-back,” with some associated blood leakage. In the future, alternative capillary options will be explored to balance between breakability and flexibility (including standard glass and Mylar-wrapped glass tubes), and the device could be modified to accept capillary tubes with a wider range of sizes and material types.

Third, hand fatigue due to the constant pressure applied during the course of collecting the FS specimen was identified as a potential concern. In the design used in the FS testing, users were required to maintain a positive-pressure grip on the push button/volume adjustment knob for the duration of blood collection, causing the noted fatigue in some users. A design adaptation to lock the push button/volume adjustment knob in place for the blood draw would resolve this issue.

Fourth, the potential for a user to incompletely insert the capillary tube into the device was identified during the user testing. In this scenario, the capillary tube would not be fully inserted at the time that FS collection occurs, which may lead to one of two potential issues. In one case, this would result in an incomplete seal and would prevent the user from having full control over the uptake and dispensing of blood. In another case, this would result in a complete—but weak—initial seal between the capillary and the rest of the device, which would make it possible for the capillary to come loose during normal device operation. This potential failure could be avoided in the future by adding visual cues that indicate when the capillary is fully inserted into the device.

Fifth, users noted that the numbers indicating the volume amounts on the dial were small and somewhat difficult to read. This can be easily resolved by enlarging the read-window and volume markers on the dial.

Lastly, the ability for small-handed users to comfortably manipulate the device was questioned and one user suggested further exaggerating the finger rest at the top of the device to help maintain grip.

There was one episode of device malfunction during the performance evaluation in which the device stopped supporting the uptake of blood by capillary action. It was determined that suds from the bleach wipe used to clean the device between donors had blocked an exterior vent hole on the device, clogging the airway between the capillary and the atmosphere. This was readily fixed by clearing out the suds, but represents a potential failure mode for future consideration.

## Conclusions

We have developed the first device capable of both collecting a large volume of FS blood (~100 μL) and dispensing that blood in variable increments as desired by the user, thus facilitating the performance of multiple POC tests (each with a different volume requirement) from a single FS sample. Our data suggests that the accuracy and precision of our prototype device compares favorably to that of a commercial P20 pipettor, while providing logistic advantages that a commercial pipettor cannot provide (collection of blood by capillary action, and variable dispensing volumes). Our device thus offers the unique possibility to reduce the patient discomfort inherent in performing multiple separate FS on one individual at one clinical visit, a testing scenario that is becoming more and more routine. While the ultimate cost of this device will depend on the final design, our goal is that the cost of the device should be less than the cost of a commercial pipettor purchased in the same country.

We recognize that operational fit, usability, and utility of our device in a given clinical setting will depend on the exact tests being performed there, as some tests have requirements for specific anticoagulants (though, as stated earlier, package inserts for some of these suggest that non-anticoagulated blood can be used if the blood can be tested quickly) and other tests have proprietary blood collection tools (some of which are, however, optional). Thus, a full understanding of the potential utility of our device in different testing scenarios will require analysis of which tests are currently used in combination in those scenarios and whether those tests are compatible regarding anticoagulant and blood collection requirements. We anticipate, however, that in the future, manufacturers who wished to ensure that their tests could be used in flexible combinations with other POC tests might include the option to use a device such as ours for FS blood collection.

There are several adjustments that could be made to facilitate future field studies of this device. First, we suggest blinding the device users to the capillary type (treated vs. untreated), because knowing that the capillary was anti-coagulant treated caused many users to relax, comfortable that the blood sample would not coagulate. Though this temperament change was not reflected in the numerical time-on-task data, users did draw the connection between the treated capillary tube and easier FS blood collection in their qualitative remarks about device ease-of-use. Second, the tradeoffs between image analysis and direct gravimetric analysis should be weighed carefully. Here, because we wanted to simulate a point-of-care setting, we used image analysis so testing did not need to stop between each aliquot to weigh the BCC. While using image analysis allowed us to gain additional knowledge on how users interact with the device in a point-of-care setting, it also prevented us from maximizing the quality of the data collected on the device’s accuracy and precision. Image analysis in future field studies could be improved by removing the crosshairs from the BCCs to allow un-impeded blood spot spreading and by photographing BCCs immediately after they were completed to try to standardize the captured blood spot color. Finally, it might be necessary to consider utilizing capillary tubes with aerosol-reducing frits for collection of blood for nucleic acid amplification testing, to avoid any carryover of nucleic acid between samples.

At the current time, testing individual patients with multiple FS POC tests in combination seems to be more common in resource-limited settings (e.g. [1–3]) than in resource-rich settings. We fully anticipate, however, that the explosion in development of FS POC tests (including tests utilizing advanced automated technology, and including molecular amplification technologies), the demonstrated acceptability of FS testing (vs e.g. oral fluid) [16, 17], and the advantages of POC testing for engaging patients who might not otherwise access medical care [18] will soon translate to the need for performance of multiple FS POC tests in individuals evaluated in resource-rich primary care settings. In short, we anticipate that our device could ultimately have high utility for facilitating and streamlining FS testing at the point of care in a diverse range of clinical settings.

## Supporting information

S1 File Study user data collection sheet. (DOCX)
