# Supplementary material for: A novel device for collecting and dispensing fingerstick blood for point of care testing
Source: PLoS One. 2017 Aug 24;12(8):e0183625. doi: 10.1371/journal.pone.0183625 (PMC5570352; doi:10.1371/journal.pone.0183625)
Supplement: S1 File — (DOCX) [file pone.0183625.s001.docx]

User Number (1-5): __________________

Donor Number (1-20): _________________

Fingerstick A or B (circle): A B

(A = uncoated capillary tube, B = NaHeparin-coated capillary tube)

**Study ID Number** (user number/donor number/fingerstick A vs B): _______/_______/_______

(record study ID # on filter paper used for absorbing dispensed blood)

Blood collection/dispensation times (to be filled out by observer):

Time when capillary tube starts to fill: _____________________

Time when capillary tube is full: _________________________

Time when dispensing is complete: ___________________________

Reason for completion of dispensing (circle):

All blood dispensed from tube blood coagulated in tube

Comments specific to this fingerstick/blood collection:

## Pre Questionnaire:

Please complete this brief questionnaire before you begin the evaluation.

1. This device is easy for me to use.

|  |  |  |  |  |
| --- | --- | --- | --- | --- |
| **Strongly Disagree** | **Disagree** | **Neutral** | **Agree** | **Strongly**  **Agree** |

Comments:

1. This device is easy for a minimally trained health worker to use.

|  |  |  |  |  |
| --- | --- | --- | --- | --- |
| **Strongly Disagree** | **Disagree** | **Neutral** | **Agree** | **Strongly**  **Agree** |

Comments:

1. Which is better for a busy nurse in a low-resource clinic? (circle)

| **A** | **B** |
| --- | --- |
| 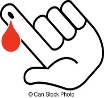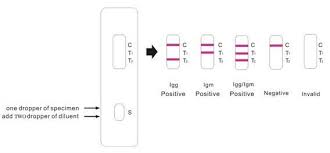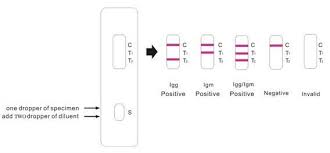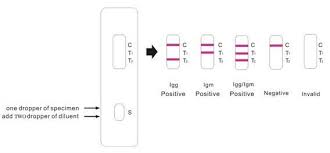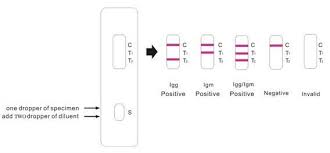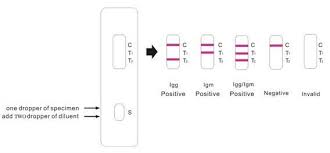 5 tests 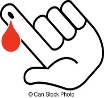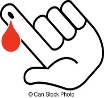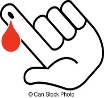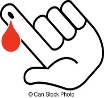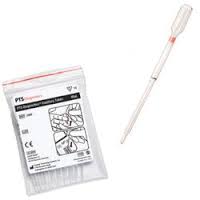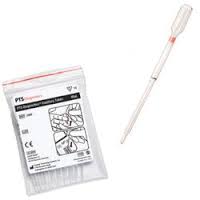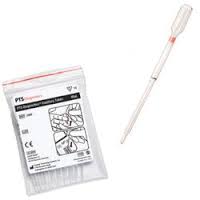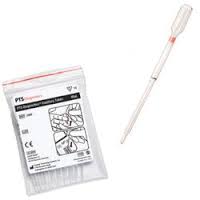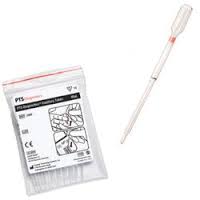 5x | 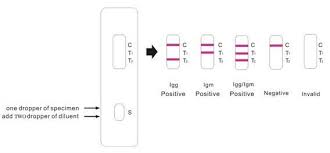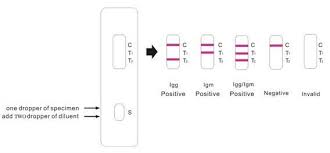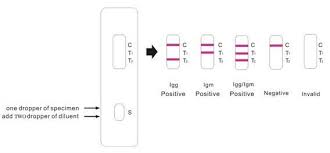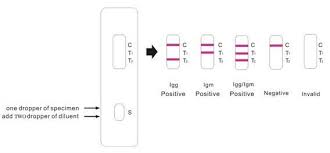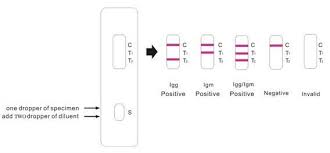 1x  5 tests 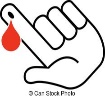 |

## Post Questionnaire:

Please complete this brief questionnaire.

1. This device is easy for me to use.

|  |  |  |  |  |
| --- | --- | --- | --- | --- |
| **Strongly Disagree** | **Disagree** | **Neutral** | **Agree** | **Strongly**  **Agree** |

Comments:

1. This device is easy for a minimally trained health worker to use.

|  |  |  |  |  |
| --- | --- | --- | --- | --- |
| **Strongly Disagree** | **Disagree** | **Neutral** | **Agree** | **Strongly**  **Agree** |

Comments:

1. Which is better for a busy nurse in a low-resource clinic? (circle)

| **A** | **B** |
| --- | --- |
| 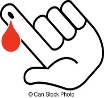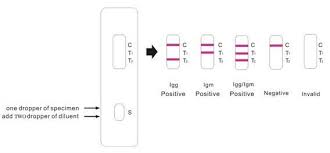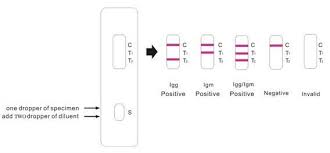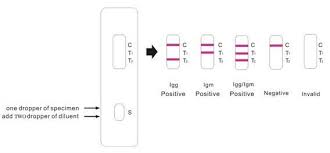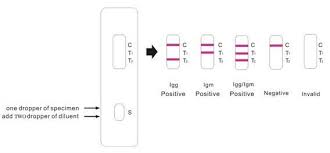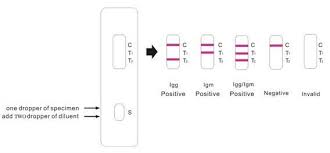 5 tests 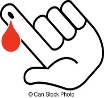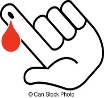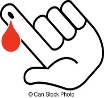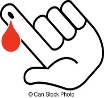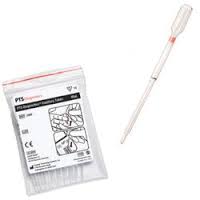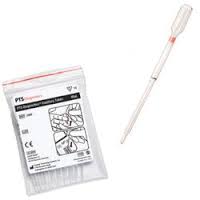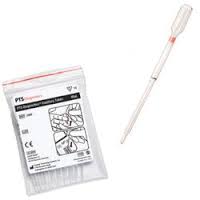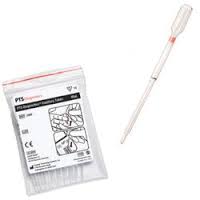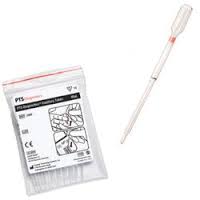 5x | 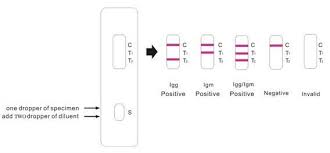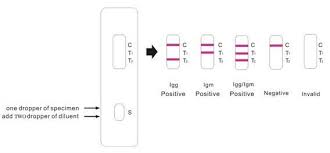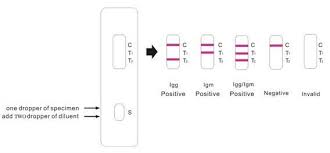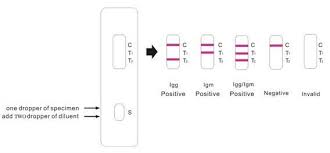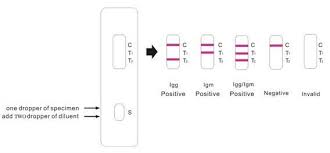 1x  5 tests 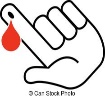 |

# Post-Evaluation Interview Guide

How easy was it to use this device? Why?

What was most difficult about using the device?

In Question #3 on the pre/post questionnaire you chose (A / B) before using the device and (A / B) after using the device. Why?

What would you change about the device for use in a low-resource clinic? Please be specific.

Based on what you know about this device, in what ways could the device be misused or caused to malfunction?

Do you have any other comments about this device?
